# Ocular and systemic vascular endothelial growth factor ligand inhibitor use and nephrotoxicity: an update

**DOI:** 10.1007/s11255-024-03990-1

**Published:** 2024-03-18

**Authors:** Dharshan Rangaswamy, Shankar Prasad Nagaraju, Mohan Varadanayakanahalli Bhojaraja, Shilna Muttickal Swaminathan, Ravindra A. Prabhu, Indu Ramachandra Rao, Srinivas Vinayak Shenoy

**Affiliations:** Department of Nephrology, Kasturba Medical College, Manipal, Manipal Academy of Higher Education, Manipal, 576104 Karnataka India

**Keywords:** Vascular endothelial growth factor, Anti-angiogenic, Drug induced nephrotoxicity, Drug induced TMA

## Abstract

Tumor growth is intricately linked to the process of angiogenesis, with a key role played by vascular endothelial growth factor (VEGF) and its associated signaling pathways. Notably, these pathways also play a pivotal “housekeeping” role in renal physiology. Over the past decade, the utilization of VEGF signaling inhibitors has seen a substantial rise in the treatment of diverse solid organ tumors, diabetic retinopathy, age-related macular degeneration, and various ocular diseases. However, this increased use of such agents has led to a higher frequency of encountering renal adverse effects in clinical practice. This review comprehensively addresses the incidence, pathophysiological mechanisms, and current evidence concerning renal adverse events associated with systemic and intravitreal antiangiogenic therapies targeting VEGF-A and its receptors (VEGFR) and their associated signaling pathways. Additionally, we briefly explore strategies for mitigating potential risks linked to the use of these agents and effectively managing various renal adverse events, including but not limited to hypertension, proteinuria, renal dysfunction, and electrolyte imbalances.

## Introduction

Pathological angiogenesis, a hallmark of tumor growth, involves the development of new blood vessels within tumors by co-opting existing ones. Numerous molecular components participate in these processes, but the central orchestrator of tumor angiogenesis is vascular endothelial growth factor (VEGF), predominantly secreted by tumor cells [1]. VEGF assumes a pivotal role as an endogenous angiogenic cytokine, serving as a central regulator of vascular growth. Its effects encompass the promotion of endothelial cell proliferation, differentiation, migration, and survival [2]. Due to its critical function, VEGF has emerged as a primary target for various therapeutic agents designed to combat cancer [3].

The human VEGF family comprises VEGF-A, VEGF-B, VEGF-C, VEGF-D, and placental growth factor, each displaying distinct binding affinities for VEGF receptors. Among these family members, VEGF-A, initially identified as a vascular permeability factor, governs blood vessel growth in both normal and pathological angiogenesis scenarios [4]. It communicates with endothelial cells through a VEGF receptor featuring tyrosine kinase activity, prompting them to proliferate and migrate. In the field of oncology, angiogenesis inhibitors targeting the VEGF ligand (anti-VEGF) or its receptors (tyrosine kinase inhibitors, TKIs) are widely employed. The Food and Drug Administration (FDA) approved the first antiangiogenic drug, bevacizumab (Avastin^®^), for clinical use in individuals with advanced colon cancer. Many of these agents have improved patient outcomes by extending overall survival and progression-free survival [1–3].

The VEGF system also plays a pivotal physiological role in maintaining various tissues and organs, including tissue repair, endometrial regeneration after menstruation, and inflammation [4]. Conditions such as pre-eclampsia, hemangiomas, and diabetic retinopathy are examples of vascular disorders with VEGF signaling pathways linked to their pathophysiology. The connections between anti-VEGF medications and various aspects of renal glomerular and tubular dysfunction, including proteinuria, hypertension, and electrolyte imbalances, are subjects of promising translational research [5, 6]. In this review, we aim to comprehensively examine and update the existing evidence, pathogenesis, early biomarkers of nephrotoxicity, and strategies to mitigate the renal impact of both ocular and systemic use of VEGF ligand inhibitors.

## Pathophysiology of VEGF inhibition

### VEGF and the eye

VEGF plays a critical role in maintaining ocular homeostasis, being produced by various cell types, including vascular endothelial cells, retinal astrocytes, retinal neurons, retinal pigmented epithelial cells, and Muller cells [7]. In pathological conditions such as hypoxia and hyperglycemia, VEGF can become overexpressed, leading to retinal angiogenesis [8]. Notably, elevated levels of vitreous and circulating VEGF have been observed in patients with both type 1 and type 2 diabetes, as well as diabetic retinopathy and diabetic nephropathy. VEGF-A has been proposed as a valuable biomarker for monitoring the progression of diabetic retinopathy [9].

The advent of VEGF inhibitors has significantly enhanced the management of various retinal ophthalmic disorders, including proliferative diabetic retinopathy, central retinal vein occlusion, diabetic macular edema, and age-related macular degeneration [10]. The intraocular administration of anti-VEGF therapy has gained widespread acceptance over the past decade [11]. Although considered a targeted therapy with minimal adverse effects and an excellent safety profile due to its specific impact on angiogenic cells with minimal harm to normal cells, it has been noted that these agents may lead to previously unknown or under-reported adverse events, particularly nephrotoxicity [12]. Anti-VEGF agents are broadly implicated in causing thrombotic microangiopathy (TMA) [13], kidney function impairment [14], or the onset or worsening of pre-existing hypertension and proteinuria [15, 16].

### VEGF and the kidney

The kidneys, highly vascularized organs, are susceptible to the ischemic effects of anti-VEGF ligands. They are both a target and a source of VEGF [17]. Vascular Endothelial Growth Factor (VEGF) plays a pivotal role in maintaining the integrity of the glomerular membrane structure and facilitating communication between podocytes and endothelial cells [18]. It serves as a vital mediator in the restoration of certain renal disorders, such as non-diabetic kidney diseases, while exerting detrimental effects in others, including diabetes and its complications [19].

In the kidney, VEGF is secreted by podocytes and tubular epithelial cells and becomes biologically active upon binding to one of the VEGF receptor tyrosine kinases (RTKs), which include VEGFR-1, VEGFR-2, and VEGFR-3. VEGFR-2 primarily mediates VEGF-A signaling. These VEGF receptors are primarily expressed in podocytes and glomerular endothelial cells (ECs) [17, 20, 21]. The interaction between VEGF produced by podocytes and VEGFR-2 on glomerular ECs, referred to as paracrine epithelial-endothelial cross-talk, is vital for the normal functioning of glomeruli and the maintenance of the glomerular barrier's integrity [22, 23].

**Fig. 1:**
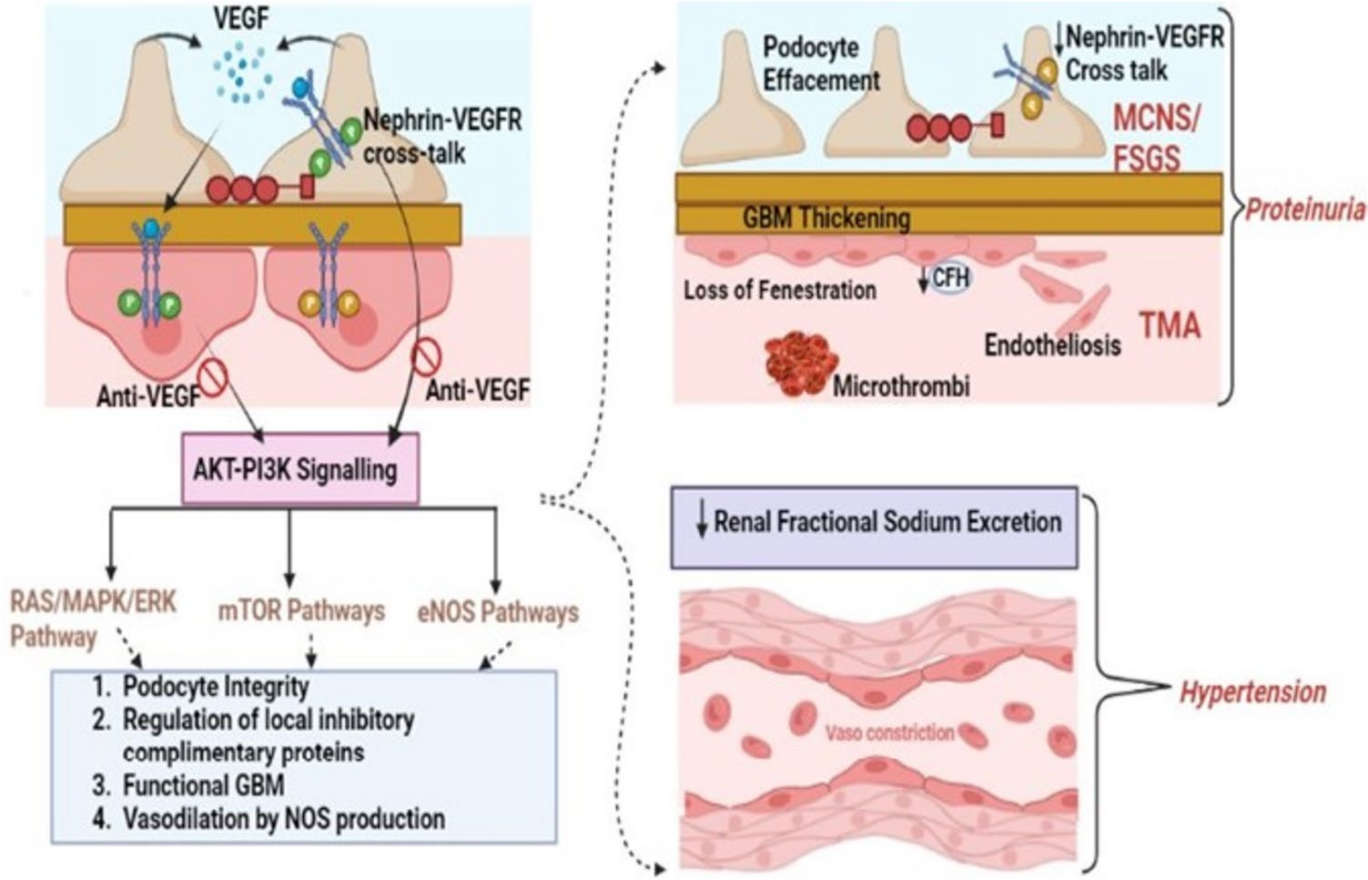
Pathogenesis of nephrotoxicity related to anti-VEGF therapy. Anti-VEGF drugs interfere with multiple downstream tyrosine kinase pathways responsible for maintaining glomerular and podocyte integrity. A consequence of this disruption is the development of podocyte effacement and glomerular basement membrane thickening, both leading to proteinuria. In addition, there is development of microthrombi, endotheliosis, and reduction in complement factors that lead to thrombotic microangiopathy and hypertension. Reduced fractional excretion of sodium also contributes to the hypertension. *VEGF* vascular endothelial growth factor, *VEGFR* vascular endothelial growth factor receptor, *AKT-PI3* phosphoinositide-3-kinase, *MAPK* mitogen-activated protein kinase, *ERK* extracellular signal-regulated kinase, *mTOR* mammalian target of rapamycin, *eNOS* endothelial nitric oxide synthase, *GBM* glomerular basement membrane, *MCNS* minimal change nephrotic syndrome, *FSGS* focal segmental glomerulosclerosis, *TMA* thrombotic microangiopathy, *CFH* complement factor-H

Within podocytes, autocrine VEGFA-VEGFR2 interaction stimulates nephrin-VEGFR crosstalk, regulating actin polymerization and stress fiber formation to maintain podocyte architecture. These effects are attributed to the fact that RTKs dimerize and undergo autophosphorylation in response to ligand binding, initiating downstream signaling pathways, including PI3 Kinase/AKT, Raf/MAPK/ERK, mTOR, and eNOS pathways [24–26]. Anti-VEGF therapy disrupts these downstream pathways, contributing to nephrotoxicity (see Fig. 1).

Numerous studies have demonstrated that VEGF treatment can reduce renal illnesses and stabilize renal function in chronic kidney disease (CKD) animals [19]. Few studies showing the benefits of VEGF therapy in experimental animal models is summarized in Table 1. VEGF exhibits a nephroprotective effect in various non-diabetic renal disorders, improving renal function and reducing renal fibrosis. This suggests that inhibiting VEGF can harm podocytes and is associated with the development of glomerulosclerosis and tubulointerstitial fibrosis [27]. Therefore, VEGF may be a critical mediator in the restoration of certain renal disorders.

**Table 1:** Experimental animal studies showing benefits of VEGF therapy in renal diseases

Author and year	Type of experimental model	Type of VEGF therapy used	Outcome of therapy
Suga et al. 2001 [28]	Severe necrotizing TMA in a rat model using antiglomerular endothelial antibodies	VEGF_121_ as subcutaneous injections	Significant reduction in glomerular necrosis and relative preservation of glomerular and tubular architecture in comparison to the control rats
Kang et al. 2001 [29]	Remant kidney(RK) model in partial nephrectomized rats leading to progressive CKD	VEGF_121_ as subcutaneous injections	As compared to controls, VEGF administered rat kidneys showed less fibrosis and increased glomerular and tubular epithelial proliferation
Kang et al. 2001 [30]	Chronic cyclosporin A (CsA) nephropathy induced in rat models through daily subcutaneous injections of CsA	VEGF_121_ as subcutaneous injections	In rats with established CsA nephropathy, VEGF resulted in control of hypertension and improved histological parameters of tubulointerstitial injury
Chade et al. 2012 [31]	Swine model—unilateral renal artery stenosis induced experimentally	Intra-renal rhVEGF-165 infused in the pigs after confirmation of stenosis after 6 weeks through multidetector -CT (MDCT) and compared with controls	VEGF infused pigs demonstrated increased renal blood flow, increased renal microvascular density and reduced renal scarring as compared to the controls
Leonard et al. [24]	Rat model—ischemia reperfusion injury leading to AKI induced by transient clamping of bilateral renal pedicles	VEGF_121_ as subcutaneous injections	The rats who received early initiation of VEGF therapy showed better preservation of microvascular density in the kidney tissue

*TMA* Thrombotic microangioapthy, *VEGF* vascular endothelial growth factor, *CKD* chronic kidney disease, *AKI* acute kidney injury

## Anti-VEGF agents and their use in clinical practice

Understanding the VEGF-VEGFR interactions in angiogenesis has unveiled a plethora of potential therapeutic targets with applications in oncology and ophthalmology. Angiogenesis and neovascularization are critical pathological processes in these fields. VEGF and its pathways are targeted at various levels, including the reduction of VEGF gene expression to mitigate its production and secretion. Among the extensively researched areas are agents commonly referred to as anti-VEGF drugs or VEGF ligand inhibitors [32]. VEGF receptors and their inhibitors, particularly tyrosine kinase inhibitors (TKIs), also find a place in the armamentarium of oncologists [33]. For this review, we will focus on anti-VEGF/VEGF ligand inhibitor agents. Commonly used agents and their indications are summarized in Table 2.

**Table 2:** Commonly used anti-VEGF agents in clinical practise and their indications

Drug	Mechanism of action	Indications
Bevacizumab	Recombinant humanized monoclonal antibody, IgG1 that binds to VEGF-A isoforms—prevents ligand-receptor interaction [34]	**USFDA approved for cancers (systemic)** Non-squamous non-small cell lung cancer Metastatic: hepatocellular, renal, cervical, ovarian, colo-rectal cancer Progressive glioblastoma **Off-label use: (intravitreal)** Diabetic macular edema Age-related macular degeneration
Aflibercept	VEGF trap, an inactive complex: glycoprotein-based molecule binds to VEGF-A and PIGF at a higher affinity than endogenous receptors [35]	**USFDA-approved (intravitreal)** Diabetic macular edema Diabetic retinopathy Wet AMD Macular edema associated with RVO Retinopathy of prematurity **Off-label (systemic)** Metastatic colorectal cancer
Ranibizumab	Recombinant, humanized monoclonal antibody: binds to all forms of VEGF-A that are biologically active—prevents binding to its receptors mainly VEGFR2 [36]	**USFDA approved (intravitreal)** Diabetic macular edema Diabetic retinopathy Wet AMD Macular edema associated with RVO Myopic choroidal neovascularization

*VEGF* Vascular endothelial growth factor, *VEGFR* vascular endothelial growth factor receptor, *USFDA* United States Food and Drug Administration, *AMD* age-related macular degeneration, *RVO* retinal vein occlusion, *PIGF* placental growth factor

## Clinical effects of anti-VEGF drugs on kidneys and current evidence

### Intravitreal anti-VEGF agents

The VEGF system plays an essential role in the pathogenesis of various ocular diseases, including proliferative retinopathies such as age-related macular degeneration, diabetic retinopathy, and central retinal vein occlusion. These diseases are characterized by ocular angiogenesis, which leads to visual impairment due to excessive VEGF activation [37, 38]. The primary goals of anti-VEGF therapy are to counteract the pathological effects of neovascularization, halt disease progression, and improve vision. Several intravitreal agents have received approval for targeting the VEGF system, including ranibizumab, aflibercept, and pegaptanib, which are commonly used. Bevacizumab, although not FDA-approved for intravitreal use, is used "off-label" for various indications by ophthalmologists. Typically, these agents are administered once monthly for 3 to 6 months, with subsequent doses adjusted based on clinical outcomes.

#### Pharmacokinetics of intravitreal anti-VEGF agents

When the FDA approved intravitreal aflibercept and ranibizumab for intravitreal use, it was based on studies indicating that the systemic concentrations achieved by these drugs administered intravitreally were nearly 200 times lower than required for maximal systemic VEGF inhibition [13]. Preclinical and clinical studies on the pharmacokinetics of anti-VEGF drugs have revealed that the blood-retinal barrier may contain neonatal Fc receptors (FcRn) that facilitate the transport of intravitreally administered drugs into the systemic circulation [39, 40]. This finding is supported by a rabbit model study in which intravitreal bevacizumab (1.25 mg) showed small amounts of the drug in the serum and the uninjected eye [41]. In a study involving patients with wet AMD, intravitreally administered anti-VEGF drugs exhibited systemic exposure (peak and trough concentrations, area under the curve) that was highest for bevacizumab, lowest for ranibizumab, and intermediate for aflibercept. Ranibizumab also did not show systemic accumulation despite repeated dosing, unlike the other two drugs, which showed systemic accumulation [42]. Studies on the pharmacokinetics of these drugs (bevacizumab, aflibercept, ranibizumab) have demonstrated that the peak systemic concentrations following intravitreal injection are equal to or exceed the half-maximal inhibitory concentration (IC50) levels for systemic VEGF inhibition [42, 43]. The peak concentration of intravitreal drugs may also vary depending on the underlying ophthalmic pathology, reflecting differences in retinal vascular permeability across different pathologies [42, 43].

#### Systemic suppression of VEGF

Evidence indicates that intravitreal anti-VEGF drugs not only achieve measurable plasma concentrations but also result in significant systemic VEGF inhibition, leading to potential systemic adverse effects. Available data suggests that aflibercept may be more potent in this regard compared to bevacizumab, with ranibizumab having the least potential for systemic VEGF inhibition [12]. Studies demonstrating systemic VEGF inhibition following intravitreal administration of these agents are summarized in Table 3. These studies reveal a notable reduction in systemic VEGF levels after intravitreal administration of the agents, with sustained suppression even one month post-injection, which may have clinical implications.

**Table 3:** Studies showing systemic VEGF inhibition by intra-vitreal anti-VEGF drugs

Author	Disease	Drug	Conclusion
Avery et al. 2014 [42]	Wet AMD	Ranibizumab bevacizumab aflibercept	All 3 agents were seen rapidly in systemic circulation after injection. Bevacizumab and aflibercept produce more profound VEGF suppression as compared to ranibizumab
Zehetner et al. 2015 [44]	Wet AMD	Aflibercept ranibizumab	89.5% of patients had profound suppression of systemic VEGF levels below MDD after 1 week. No significant suppression with ranibizumab
Yoon et al. 2016 [45]	CNV, DR, AMD, BRVO	Bevacizumab ranibizumab	Bevacizumab was associated with a significant reduction in VEGF levels after one day, one week and one month compared to no significant changes with ranibizumab
Rogers et al. (2018) [46]	nAMD	Bevacizumab ranibizumab	Systemic VEGF levels are suppressed to a greater extent by bevacizumab compared to ranibizumab. This suppression is diminished with treatment discontinuation of 3 months
Hirano et al. 2018 [47]	Diabetic macular edema	Bevacizumab aflibercept ranibizumab	Both bevacizumab and aflibercept are associated with significantly reducing systemic VEGF levels at weeks 1 and 4. No significant effects were seen with ranibizumab

*DME* Diabetic macular edema, *BRVO* branch retinal vein occlusion, *AMD* age-related macular degeneration, *VEGF* vascular endothelial growth factor, *CNV* choroidal neovascularization, *DR* diabetic retinopathy, *MDD* minimum detectable dose

#### Clinical effects of intravitreal VEGF blockade on the kidneys

Despite pharmacokinetic studies suggesting sustained systemic VEGF suppression, the translation of this into clinical outcomes has yielded mixed results. Studies in monkeys have demonstrated aflibercept’s presence in the glomeruli one week after intravitreal injection [48]. Evidence for renal effects includes the development of de novo hypertension [49, 50], worsening of pre-existing hypertension [51, 52], acute kidney injury [53], and proteinuria [51, 53]. Additionally, there are over 32 biopsy-proven cases of glomerular diseases temporally associated with intravitreal anti-VEGF injections, including worsening or relapse of pre-existing glomerular pathologies and the development of de novo glomerular diseases, including collapsing FSGS and TMA [15]. Table 4 summarizes studies showing these drugs' potential renal effects. On the other hand, a few studies have shown no effect on proteinuria or GFR with the use of intravitreal anti-VEGF agents [54–56]. Studies by Kameda et al. and Glassman et al. included diabetic CKD patients and patients with pre-existing albuminuria, with both studies suggesting no changes in GFR, blood pressure, or albuminuria during follow-up of patients receiving anti-VEGF agents [54, 55].

**Table 4:** Studies showing renal effects of intravitreal anti-VEGF inhibitors

Author	Disease	Drug	Renal effect
Scott et al. 2007 [57]	DME	Bevacizumab	Elevated blood pressure and worsening renal functions at 24 weeks
Rasier et al. 2009 [50]	Wet AMD	Bevacizumab	Elevated systolic and diastolic blood pressure in both non-hypertensives (at week 3) and hypertensives (week 1, 3, 6)
Shah et al. 2019 [52]	Diabetic retinopathy	Bevacizumab aflibercept ranibizumab	Higher blood pressure is associated with receiving higher number of anti-VEGF injections
Hanna et al. [13, 14, 16, 58, 59]	Diabetic retinopathy, DME, CRVO	Bevacizumab aflibercept ranibizumab	Worsening proteinuria, hypertension & eGFR. Chronic TMA, FSGS on renal biopsy

*DME* Diabetic macular edema, *CRVO* central retinal vein occlusion, *AMD* age-related macular degeneration, *VEGF* vascular endothelial growth factor, *eGFR* estimated glomerular filtration rate, *TMA* thrombotic microangiopathy, *FSGS* focal segmental glomerulosclerosis

### Systemic agents

Most of the experience has been with bevacizumab, a humanized monoclonal antibody (IgG1) against VEGF-A, which prevents the activation of VEGFR. The VEGF family includes at least five known ligands: VEGF-A, VEGF-B, VEGF-C, VEGF-D, and placenta growth factor. These ligands, especially VEGF-A, bind to receptors such as VEGFR-1 (Flt-1), VEGFR-2 (KDR/Flk-1), and VEGFR-3, along with the coreceptor neuropilins, to induce normal and tumor-associated angiogenesis [60]. In normal kidneys, VEGF is continually expressed and secreted by podocytes. Locally produced VEGF acts on VEGF receptors located on glomerular and peritubular endothelium and mesangial cells, maintaining normal glomerular function and the integrity of the glomerular basement membrane (GBM) [61]. Interfering with the paracrine function of VEGF alters local glomerular physiology, resulting in clinical and pathological manifestations of kidney damage, such as proteinuria, nephrotic syndrome, acute kidney injury, thrombotic microangiopathy (TMA), and hypertension observed with the administration of VEGF ligand inhibitors [61].

**Fig. 2:**
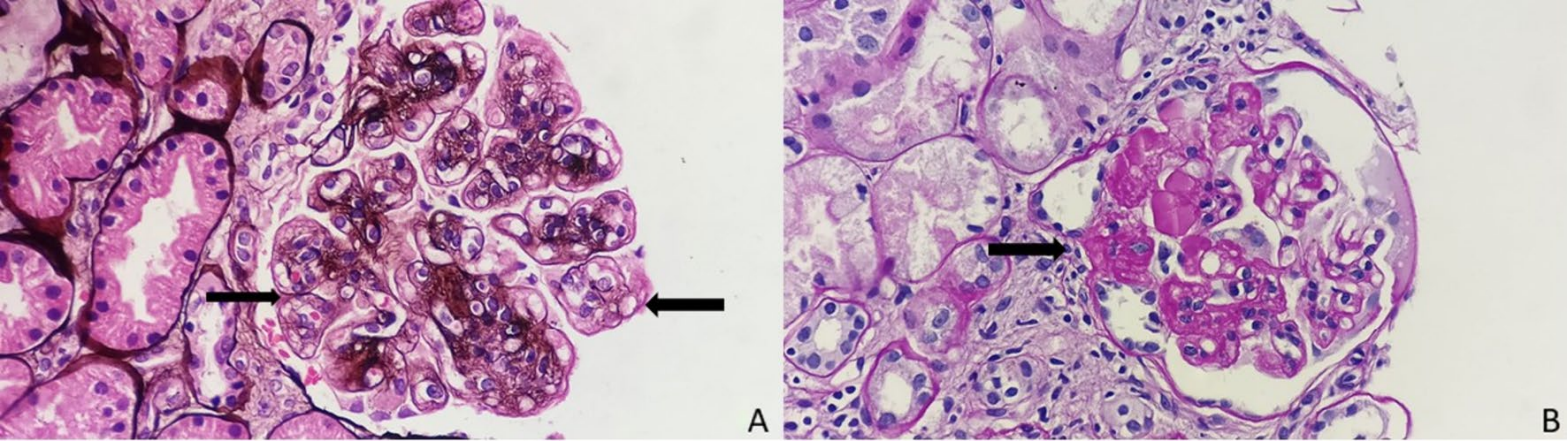
**A** Glomerulus with features of chronic thrombotic microangiopathy. Diffuse and global duplication of glomerular basement membranes (arrows) is noted with segmental mesangial matrix expansion (PASM stain; 400×); **B** glomerulus showing segmental sclerosis with hyalinosis (arrow; PAS stain; 400×). Both images are representative images of lesions seen in patients with renal effects of anti-VEGF therapy and not actual patient images

Kidney biopsies from patients treated with VEGF-ligand inhibitors have demonstrated TMA, focal segmental glomerulosclerosis, collapsing glomerulopathy (especially among patients with a history of pamidronate use), and occasional case reports of cryoglobulinemic, immune complex, and proliferative glomerulonephritis (more commonly associated with tyrosine kinase inhibitors, TKIs) [62–64] (Fig. 2). Complement activation can also be observed in TMA due to reduced complement factor H (CFH) and fibrin microthrombi in glomerular capillaries [65]. Although not mutually exclusive, VEGF-ligand inhibitors are associated with an increased incidence of TMA, while TKIs exhibit increased podocytopathies [66]. Furthermore, anti-VEGF therapy induces vascular resistance by reducing nitric oxide production and renal fractional sodium excretion (FENa), contributing to volume-dependent hypertension [67, 68].

The renal effects seem to be dose-dependent, with increasing nephrotoxicity observed when doses ≥ 10 mg/kg/dose (high dose) are used. In patients receiving ≤ 7.5 mg/kg/dose (low dose) bevacizumab, the incidence of proteinuria and hypertension ranged from 21 to 41% and between 2.7 and 32%, respectively. In the high dose group, an increased incidence of 22 to 63% for proteinuria and 17.6 to 36% for hypertension was observed [69]. A meta-analysis of clinical trials involving bevacizumab use in solid tumors identified an incidence of 7.9% for high-grade (grade 3 or 4) hypertension [70]. Nephrotoxicity is more frequent in patients with pre-existing renal disease, a diagnosis of renal cell carcinoma, and concurrent use of chemotherapeutic agents such as cisplatin and bisphosphonates like pamidronate [65, 69]. The package insert for bevacizumab treatment recommends temporarily withholding treatment for patients with new-onset moderate to severe proteinuria and those with uncontrolled severe hypertension. The insert also recommends permanently discontinuing treatment with bevacizumab in patients who develop nephrotic syndrome or a hypertensive crisis. Importantly, the nephrotoxic effects can be irreversible despite discontinuing the therapy [71]. A study by Li Y et al. and Roviello G et al. positively linked sorafenib-induced hypertension and ramucirumab-induced hypertension to antitumor response [72, 73]. No such predictive response was observed in the meta-analysis of seven phase 3 RCTs with bevacizumab use across multiple tumor types [74].

## Predicting and mitigating renal effects of anti-VEGF drugs

No established society guidelines or studies have investigated the management of renal complications of anti-VEGF drugs or recommendations regarding a class of antihypertensives to manage proteinuria and secondary hypertension. However, prior experience from animal studies, observational studies, and case reports has shed light on potential preventive and therapeutic measures.

1. Identifying patients at risk: patients with baseline comorbid conditions like hypertension, diabetes, chronic kidney disease (CKD), and proteinuria are likely to experience an exacerbation of underlying kidney disease when using these agents [16, 50]. Hence, these patients may be considered “at risk” and should be planned for close monitoring.
2. Monitoring for renal side effects during therapy: once at-risk patients are identified, regular monitoring is crucial after receiving anti-VEGF therapy. Active blood pressure monitoring and albuminuria measurement, before and after initiation of therapy, are of prime importance [75]. Any worsening of hypertension, proteinuria, or creatinine levels may indicate a renal effect, and the patient should undergo further assessment, including a renal biopsy. A detailed discussion should ensue with the patient, explaining the risks and benefits of continuing VEGF-ligand inhibitors in the presence of ongoing renal injury. Discontinuation of bevacizumab can reduce proteinuria and improve hypertension control. The use of angiotensin-converting enzyme inhibitors or angiotensin receptor blockers, as in the general population, to reduce proteinuria and lower intra-glomerular pressure, may not replicate similarly in patients on anti-VEGF therapy. Siddique et al. demonstrated inhibition of VEGF ligand and VEGFR expression in the myocardium of rats exposed to enalapril or candesartan [76].
3. Biomarkers of nephrotoxicity: chebotareva and colleagues examined various urinary biomarkers and their ability to predict nephrotoxicity in patients exposed to anti-VEGF ligands. Elevated urinary biomarkers at the end of the first and second week following ranibizumab/bevacizumab/aflibercept administration predicted the clinical occurrence of nephrotoxicity at week 8 with over 65% sensitivity and specificity. Urinary Neutrophilic Gelatinase-Associated Lipocalin (NGAL), expressed significantly from the distal segment of the nephron; urinary Kidney Injury Molecule 1 (KIM-1), a proximal tubular transmembrane protein; Hypoxia Inducible Factor-1α (HIF-1α), reflecting rarefaction of peritubular capillaries and renal tissue hypoxia; and Nephrin, indicating a break in the glomerular filtration barrier, were elevated following a single dose of anti-VEGF ligand, suggesting injury across different nephron segments [77].
4. Treatment of renal side effects: in patients identified as potentially having developed anti-VEGF therapy-induced renal side effects such as proteinuria, hypertension, or worsening creatinine, no proven therapies can directly facilitate these changes. Managing hypertension and proteinuria with angiotensin-converting enzyme inhibitors or angiotensin receptor blockers (ACEi/ARBs) may be a prudent approach. However, reducing the dose of the anti-VEGF agent, using alternate agents, or stopping drug therapy may be necessary to prevent further damage [12]. As stated previously, nephrotoxicity of bevacizumab is dose-dependent and is more often observed when the dose is ≥ 10 mg/kg/dose, and nephrotoxicity may be irreversible despite stopping the drug.

## Conclusion and future directions

Anti-angiogenic therapy stands as a promising and innovative approach for addressing conditions reliant on angiogenesis. Specifically, inhibitors targeting vascular endothelial growth factor (VEGF) have emerged as powerful tools in managing angiogenesis-dependent disorders like cancer and diabetic retinopathy, due to their capacity to inhibit angiogenesis. As VEGF plays a crucial role in maintaining renal homeostasis, the use of VEGF ligand inhibitors has been associated with various renovascular conditions. These conditions manifest as proteinuria, hypertension, nephrotic syndrome, reduced glomerular filtration rate (GFR), and thrombotic microangiopathy (TMA). The identification of individuals at risk for nephrotoxicity, the utilization of urinary biomarkers as indicators of renal injury, and the implementation of strategies to minimize exposure to high-dose VEGF ligand inhibitors collectively contribute to improving renal outcomes.
